# Supplementary material for: Effect of storage condition on the physico-chemical properties of corn–wheat starch/zein edible bilayer films
Source: R Soc Open Sci. 2020 Feb 12;7(2):191777. doi: 10.1098/rsos.191777 (PMC7062101; doi:10.1098/rsos.191777)
Supplement: Electronic Supplementary Material-The application of the C-W/Z bilayer films [file rsos191777supp1.doc]

Electronic Supplementary Material

**The application of the C-W/Z bilayer films**

By studying the changes in the peroxide value of the packaging content in different storage environments, the overall performance of C-W/Z bilayer film oil package (BOP) was evaluated. And through a comparative study with commercial film oil package (COP), it can be judged whether the BOP meets the actual application requirements.

**Section I. Materials and methods**

**①. Preparation of the oil packages**

Heat seal the two sides of the C-W/Z bilayer film with a diameter of 120 mm after folding, then put 3 ml of fresh soybean oil into it, and heat seal it again to make the BOP. The heat-sealing voltage, vacuum time, heat-sealing time and cooling time were 24 V, 5.0 s, 2.0 s and 4.0 s, respectively. Similar to the preparation method of the BOP, the commercial films (BOPP-PE, Nanjiecun Co., Ltd., Henan, China) were used to prepare COP.

**②. Storage of the BOP and COP**

The BOP and COP were stored for 20, 40, 60, 80, 100,120 d under freezing (-17°C), refrigeration (4°C), and room temperatures (25°C; RH: 43%, 54%, 65%). The POV of the soybean oil was measured every 20 days to evaluate the quality change of the soybean oil within 120 days.

**③. Drop tests**

The drop tests of the BOP refer to part 6.5.11 of the “Chinese National Standard GB/T 21302-2007”. A smooth and hard test surface was used, and the BOP was dropped from 1000 mm along the parallel and vertical directions, respectively. Observe whether the BOP was leaking oil. Each group of experiments was repeated 5 times.

**④.** **Compression tests**

The compression tests of the BOP refer to part 6.5.10 of the “Chinese National Standard GB/T 21302-2007”. Two plates were placed on the TAXT-plus texture analyser, and a filter paper with a diameter of 120 mm was placed on the plate. Then, the BOP was placed on the filter paper and kept for 60 s under the force of 100 N to observe whether the BOP was broken and whether there were oil droplets remaining on the filter paper. Each group of experiments was repeated 5 times.

**Section II. Results and discussions**

**①. Appearance of BOP and COP**


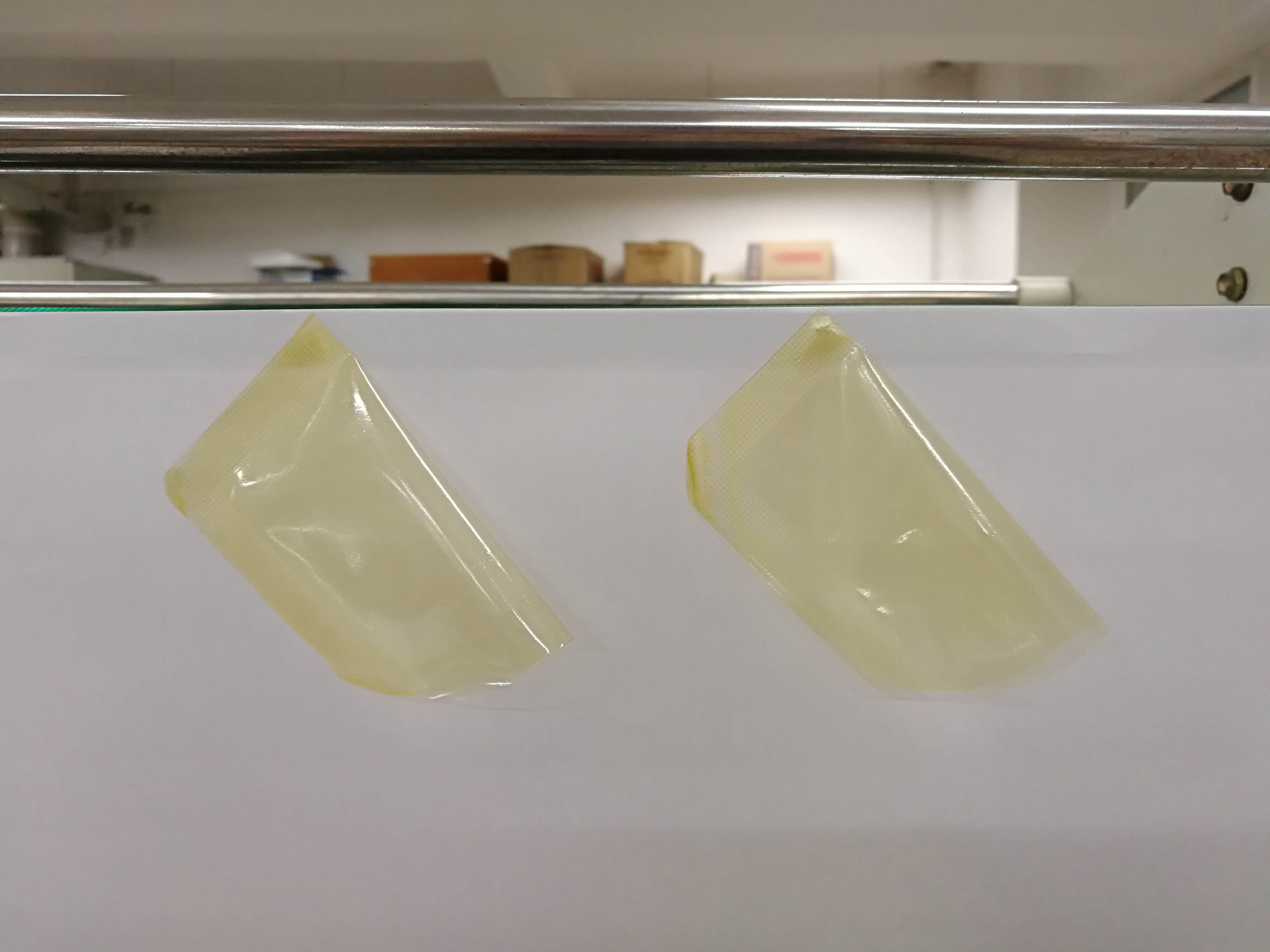

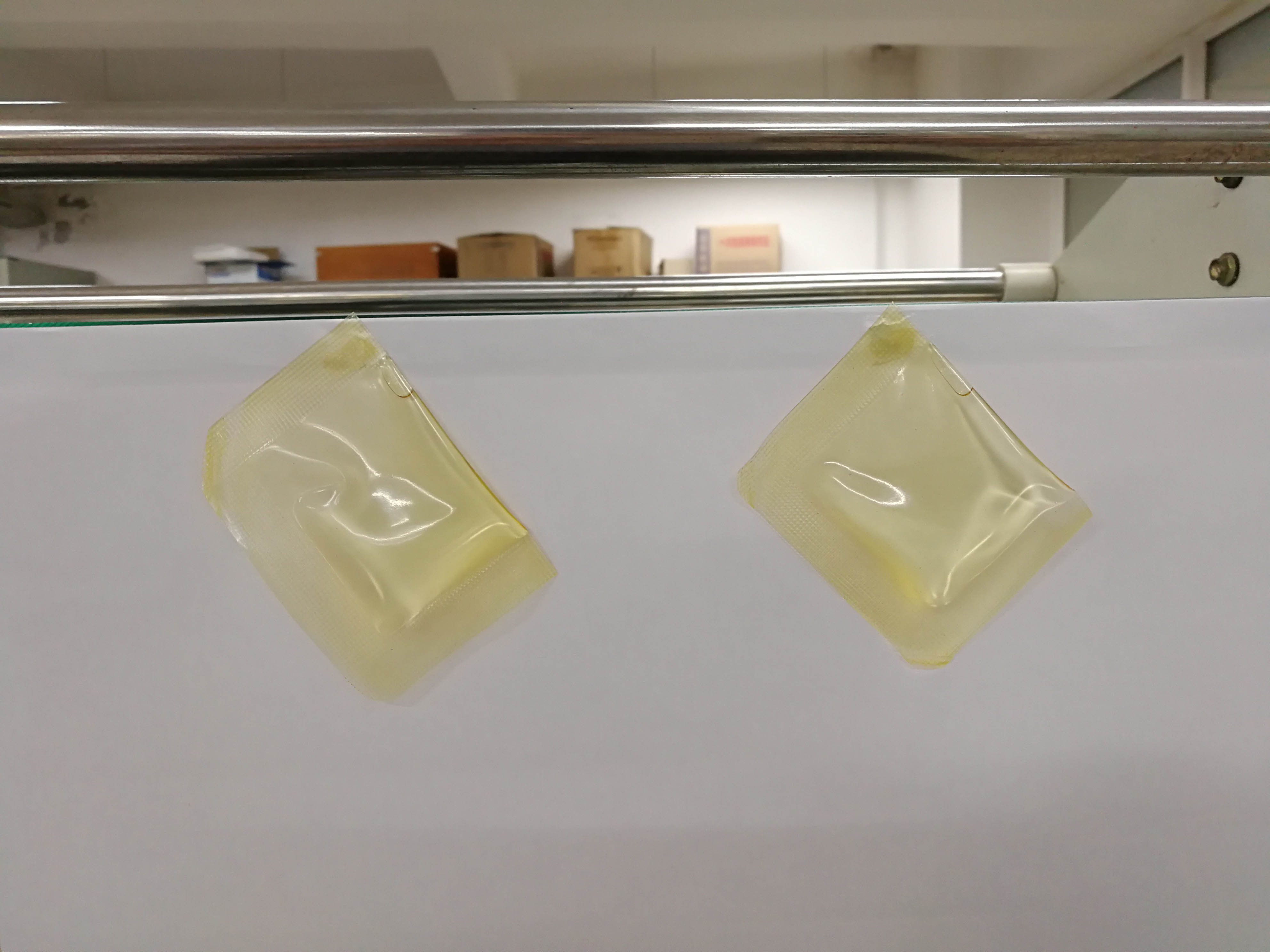


(a) (b)


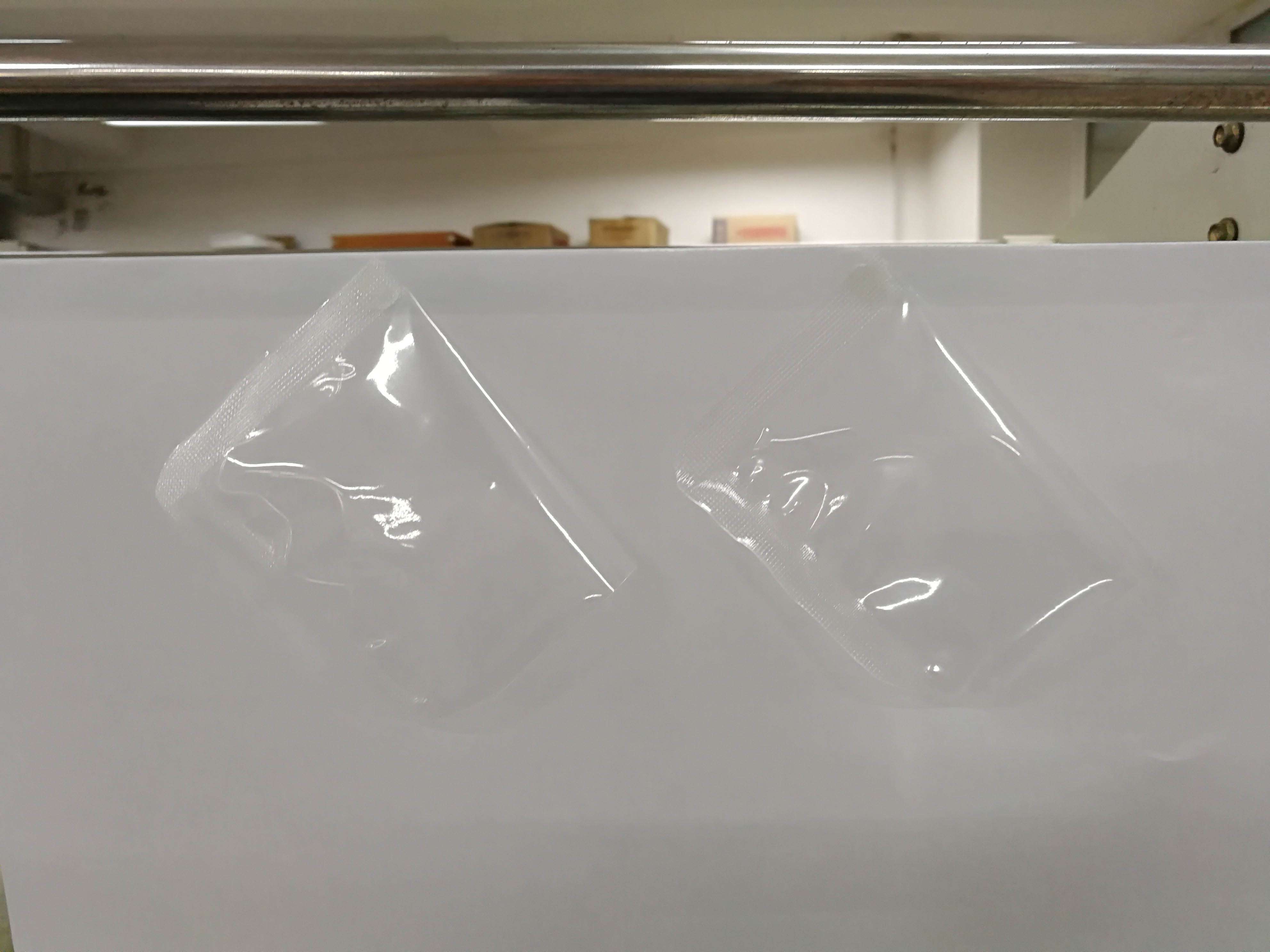

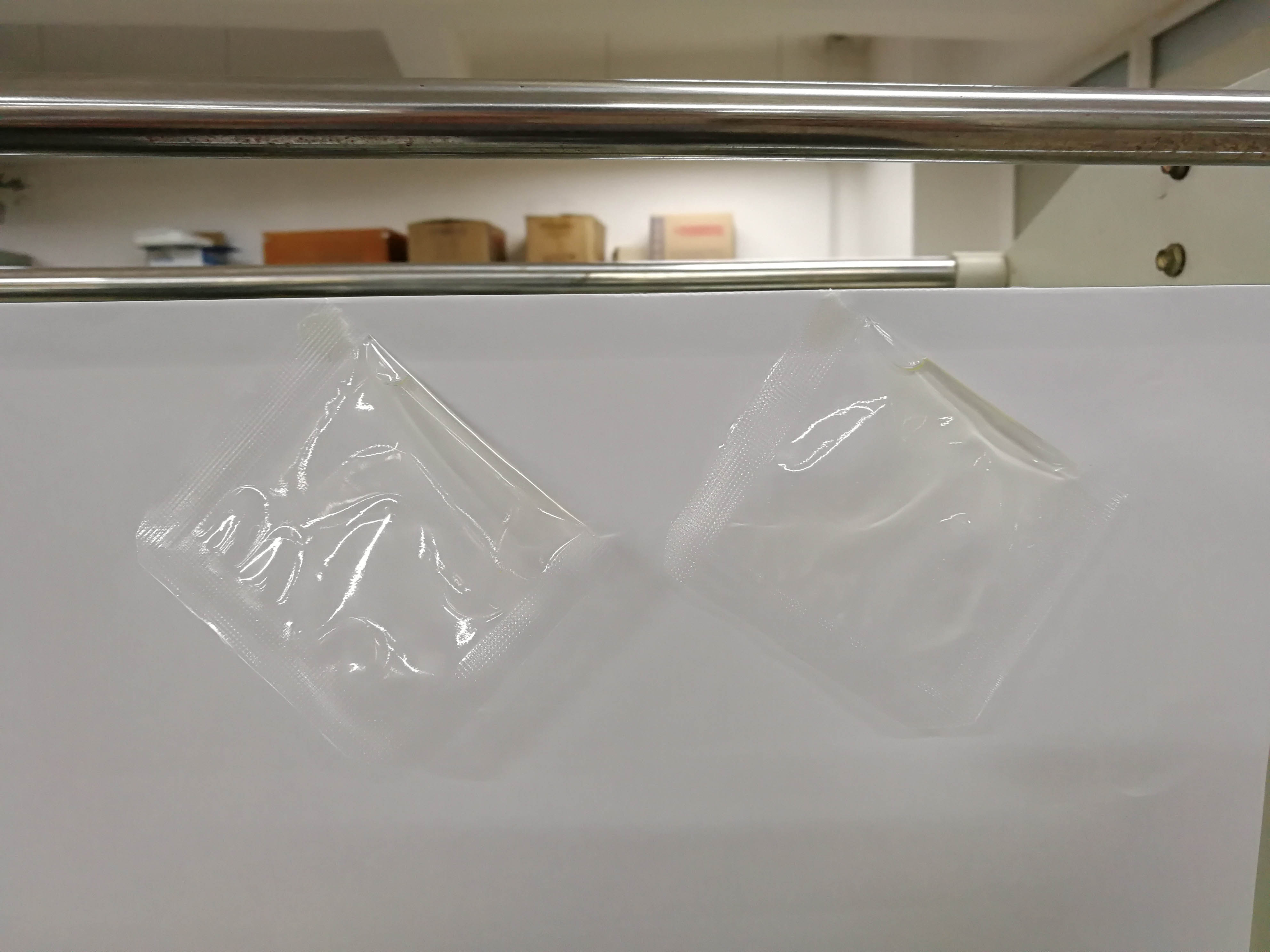


(c) (d)

Fig. S1 Appearance of the films and oil packages: (a) the C-W/Z bilayer films at 0 d; (b) the BOP at 0 d. (c) the commercial films at 0 d; (d) the COP at 0 d.

Film colour is an important factor in terms of consumer acceptance of both edible and inedible ﬁlms in packaging applications. Fig. S1 shows the appearance of the films and oil packages at 0 d. The colour difference between the C-W/Z bilayer films and the commercial films may be due to the different components , plasticizer addition and preparation methods .

**②. Comparative analysis on the oxygen barrier properties of BOP and COP**

Table S1 shows the changes in the POV of the soybean oil in BOP and COP under different storage conditions, respectively. Under the same storage condition and the same storage time, the POV of the soybean oil in BOP was basically equal to or lower than that in COP, indicating that the BOP had certain application value. In addition, the initial POV of soybean oil was 1.46 mmol/kg. After 120 d of storage, the POV of soybean oil under -17°C and 4°C were significantly lower than that under 25°C (P < 0.05) and met the standard of grade I soybean oil (5.00 mmol/kg). This is because the internal structure of the films became compact under low temperature, which hindered the transfer of oxygen molecules; on the other hand, low temperature inhibited the lipid oxidation of the oil.

Table S1 Changes in POV of the soybean oil during storage（mmol/kg）

| Storage  conditions | POV_20 d_ | POV_40 d_ | POV_60 d_ | POV_80 d_ | POV_100 d_ | POV_120 d_ |
| --- | --- | --- | --- | --- | --- | --- |
| 17 ^o^C_BOP_ | 2.71±0.03^a, AB^ | 2.71±0.10^a, A^ | 3.20±0.05^b, A^ | 3.42±0.12^c, AB^ | 3.65±0.20^d, AB^ | 4.10±0.14^e, A^ |
| -17 ^o^C_COP_ | 2.91±0.06^a, ABC^ | 2.94±0.07^a, AB^ | 3.87±0.09^b, B^ | 4.00±0.11^bc, CD^ | 4.14±0.13^c, BC^ | 4.19±0.17^c, AB^ |
| 4 ^o^C_BOP_ | 2.61±0.14^a, A^ | 3.06±0.09^ab,AB^ | 3.34±0.17^b, A^ | 3.21±0.64^b, A^ | 3.45±0.15^b, A^ | 4.25±0.12^c, AB^ |
| 4 ^o^C_COP_ | 2.79±0.40^a, ABC^ | 2.89±0.08^a, AB^ | 3.83±0.20^b, B^ | 4.21±0.15^bc, D^ | 4.20±0.27^bc, BC^ | 4.53±0.17^c, BC^ |
| RH:43%_BOP_ | 3.13±0.17^a, CDE^ | 3.12±0.13^a, AB^ | 3.71±0.26^b, B^ | 4.68±0.17^c, E^ | 4.66±0.29^c, C^ | 4.70±0.57^c, CD^ |
| RH:43%_COP_ | 3.40±0.27^a, DE^ | 4.06±0.39^b, D^ | 5.03±0.32^c, D^ | 7.04±0.09^d, G^ | 8.67±0.18^e, E^ | 9.04±0.09^e, F^ |
| RH:54%_BOP_ | 2.55±0.08^a, A^ | 2.92±0.06^b, AB^ | 3.31±0.06^c, A^ | 3.75±0.05^d, BC^ | 3.76±0.27^d, AB^ | 4.95±0.18^e, D^ |
| RH:54%_COP_ | 3.35±0.10^a, DE^ | 3.99±0.22^b, CD^ | 4.80±0.44^c, D^ | 7.12±0.22^d, G^ | 8.73±0.64^e, E^ | 9.34±0.11^e, FG^ |
| RH:65%_BOP_ | 3.03±0.13^a, BCD^ | 3.46±0.31^b, BC^ | 4.37±0.03^c, C^ | 5.63±0.09^d, F^ | 6.76±0.03^e,D^ | 7.15±0.17^f, E^ |
| RH:65%_COP_ | 3.50±0.35^a, E^ | 5.05±0.84^b, E^ | 4.67±0.12^b, CD^ | 6.94±0.09^c,G^ | 9.33±0.41^d, F^ | 9.63±0.10^d, G^ |

Different lower-case and upper-case letters in the table indicate significant differences (P<0.05) in the same row and column, respectively.

**③. Results of BOP drop tests**

The drop test has been done to verify whether the BOP meets the requirements for convenient food transportation. , The evaluation results of drop test of BOP are shown in Table S2. The BOP was dropped freely twice at a height of 1000 mm, and it was found that the BOP was not broken and no oil leakage occurred. Fig. S2 shows the sample of BOP before and after dropping. It can be seen that the appearance shape of BOP did not changed significantly after dropping. The test results met the requirements for convenient food transportation.


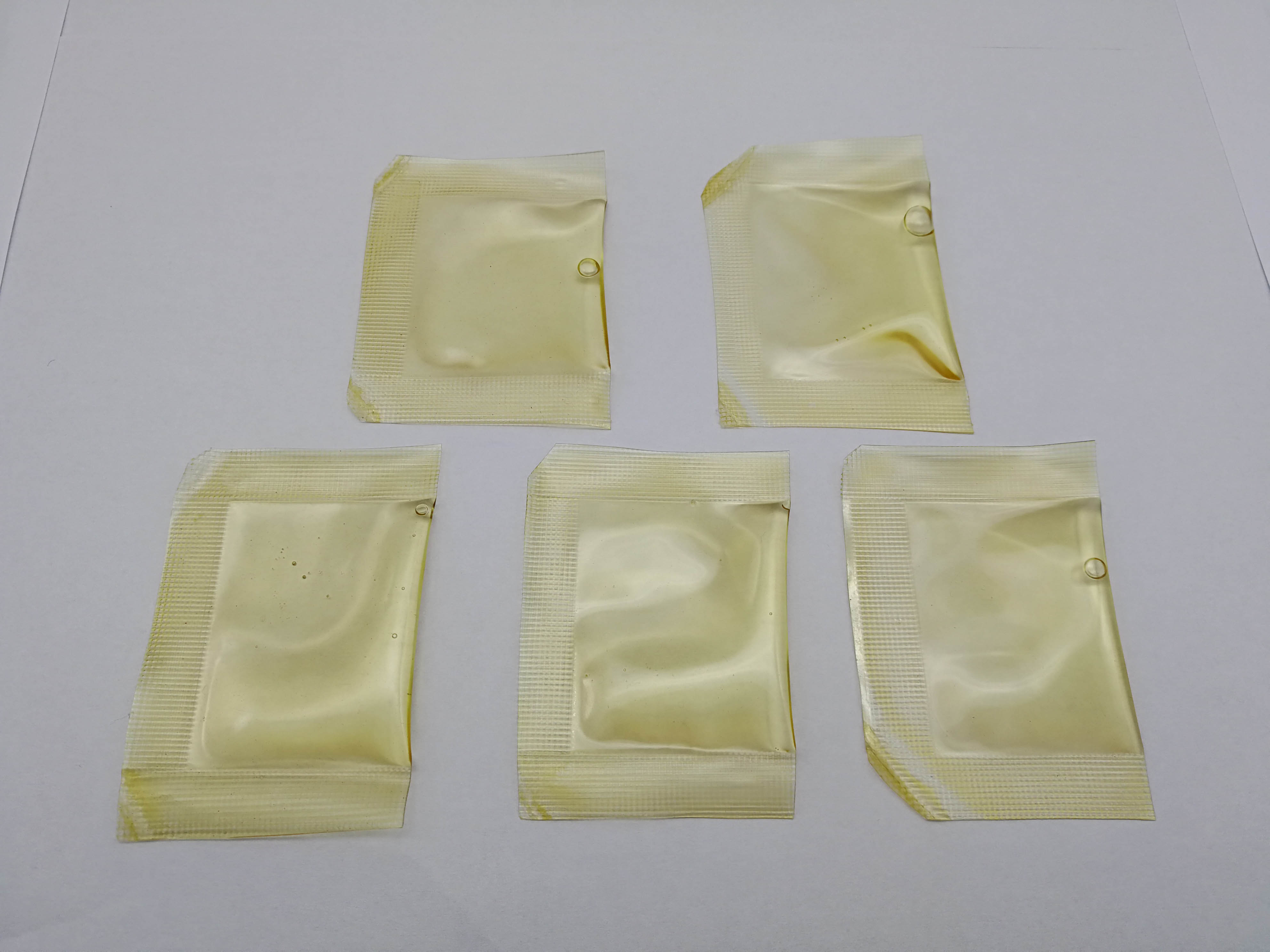

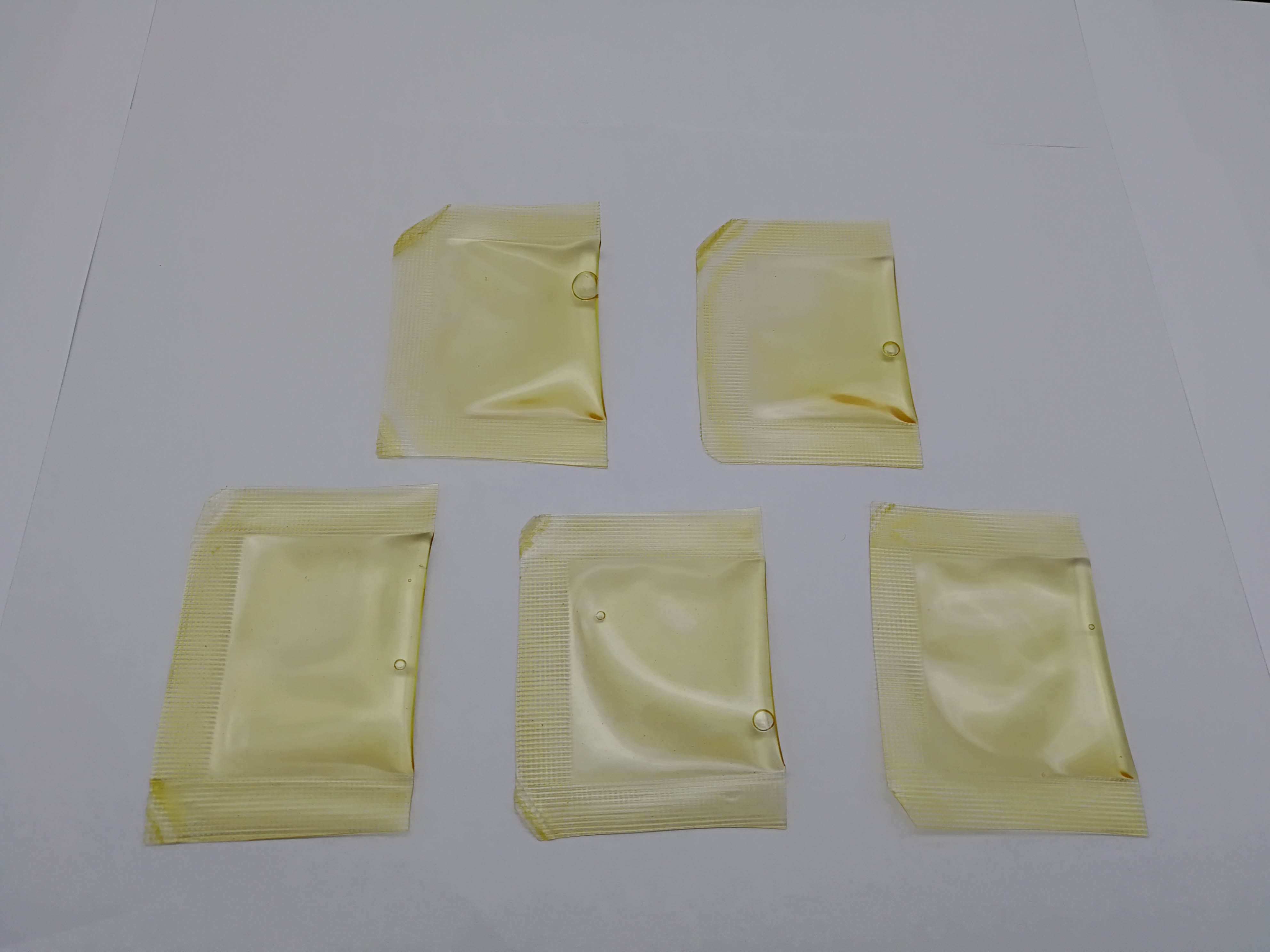


(a) (b)

Fig. S2 Photographs of the C-W/Z bilayer film oil packages in drop tests: (a) the BOP before the drop; (b) the BOP after the drop.

Table S2 Drop test results of the BOP

| Total mass (g) | drop height (mm) | Test requirements | Results |
| --- | --- | --- | --- |
| <100 | 1000 | No cracking or leakage | Qualified |

**④. Results of BOP compression tests**

The compression test of the BOP has been done to evaluate whether it can meet the stacking requirements by testing the static pressure resistance of a single independent package.

Table S3 shows the results of the BOP compression tests. After the BOP was subjected to a static pressure of 100 N for 60 s, the samples and the filter papers were shown in Fig. S3. It was observed that there was no rupture of the BOP and no oil droplets remained on the filter paper. Therefore, the BOP met the requirements of food internal packaging stacking.

Table S3 Compression test results of the BOP

| Total mass (g) | Load (N) | Test requirements | Results |
| --- | --- | --- | --- |
| <30 | 100 | No cracking or leakage | Qualified |


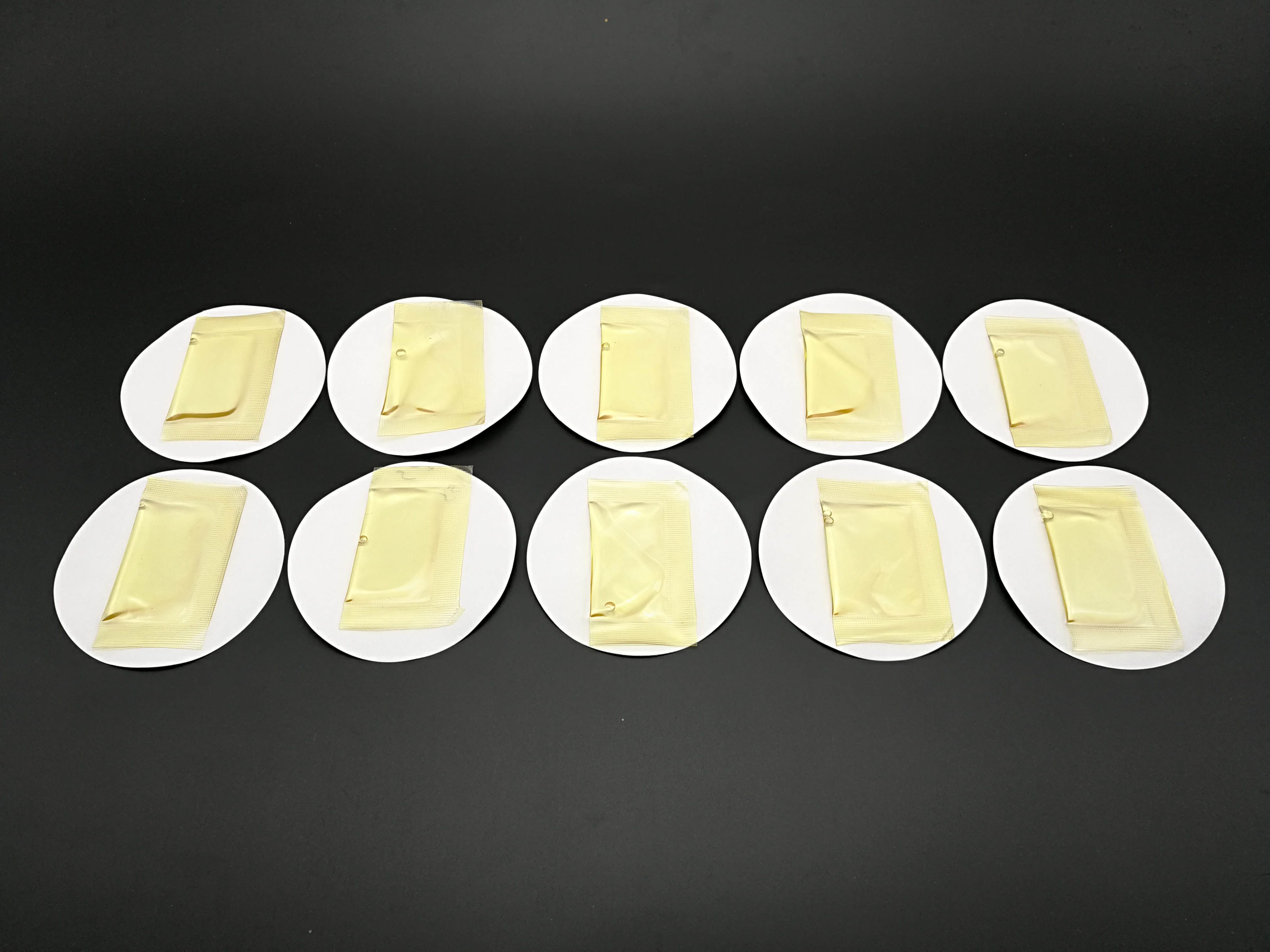

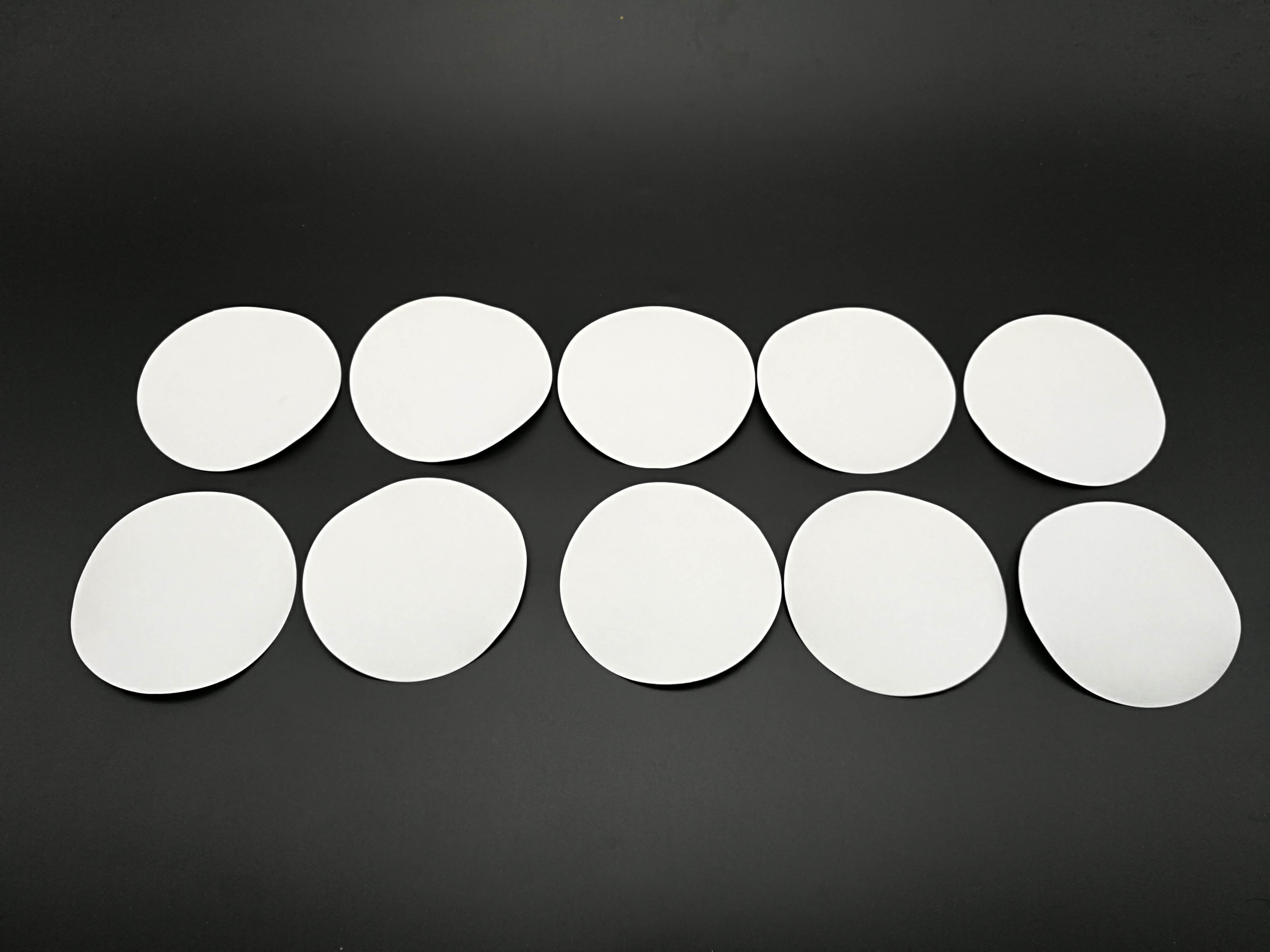


(a) (b)

Fig. S3 Photographs of compression test results: (a) the BOP after compression test; (b) filter papers in the BOP compression test.

**Section III. Conclusion**

During storage, the POV of the soybean oil in the BOP was equal to or lower than the POV in COP. Furthermore, the results of drop tests and compression tests showed that the BOP met the requirements of actual transportation and stacking. The C-W/Z bilayer films had great potential for practical application in oil package production industry.

**References**

1. Su JF, Yuan XY, Huang Z, Wang XY, Lu XZ, Zhang LD, Wang SB. 2012 Physicochemical properties of soy protein isolate/carboxymethyl cellulose blend films crosslinked by Maillard reactions: Color, transparency and heat-sealing ability. *Mater. Sci. Eng. C.* **32**, 40-46. (https://doi.org/10.1016/j.msec.2011.09.009)

2. Borneo R, Alba N, Aguirre A. 2016 New films based on triticale flour: Properties and effects of storage time. *J. Cereal Sci.* **68**, 82-87. (DOI: 10.1016/j.jcs.2016.01.001)

3. Pérez L, Piccirilli G, Delorenzi N, Verdini R. 2016 Effect of different combinations of glycerol and/or trehalose on physical and structural properties of whey protein concentrate-based edible films. *Food Hydrocolloids*. **56**, 352-359. (DOI: 10.1016/j.foodhyd.2015.12.037)
